# Pseudogenization of the *Humanin* gene is common in the mitochondrial DNA of many vertebrates

**DOI:** 10.24272/j.issn.2095-8137.2017.049

**Published:** 2017-07-18

**Authors:** Ian S. Logan

**Affiliations:** 22 Parkside Drive, Exmouth, Devon, UK

**Keywords:** mtDNA, Humanin, Pseudogenization, NUMT

## Abstract

In the human the peptide Humanin is produced from the small *Humanin* gene which is embedded as a gene-within-a-gene in the 16S ribosomal molecule of the mitochondrial DNA (mtDNA). The peptide itself appears to be significant in the prevention of cell death in many tissues and improve cognition in animal models. By using simple data mining techniques, it is possible to show that 99.4% of the human *Humanin* sequences in the GenBank database are unaffected by mutations. However, in other vertebrates, pseudogenization of the Humanin gene is a common feature; occurring apparently randomly in some species and not others. The persistence, or loss, of a functional *Humanin* gene may be an important factor in laboratory animals, especially if they are being used as animal models in studies of Alzheimer's disease (AD). The exact reason why *Humanin* underwent pseudogenization in some vertebrate species during their evolution remains to be determined. This study was originally planned to review the available information about *Humanin* and it was a surprise to be able to show that pseudogenization has occurred in a gene in the mtDNA and is not restricted solely to chromosomal genes.

## INTRODUCTION

The peptide Humanin was first described in 2001 (Hashimoto et al., 2001) when it was observed during a study on Alzheimer's disease (AD) that the death of cells was prevented by the presence of the peptide. A further paper from the same research group (Terashita et al., 2003) described how the sequence of the peptide appeared to be produced by a hitherto unrecognised gene-within-a-gene in the *MT*-*RNR2* gene of the human mitochondrial DNA (mtDNA). Subsequently, Bodzioch et al. (2009) described that the *Humanin* gene was responsible for the production of Humanin, but also suggested that the peptide might be produced from chromosomal DNA as sequences very similar to mitochondrial *Humanin* could be found in the fragmentary copies of the mtDNA that exist in nuclear chromosomal DNA. This report also mentioned that sequences similar to the *Humanin* gene, as found in the human, are '*relatively well*-*conserved in mtDNA*, *where they can be traced down the whole evolutionary tree*'. Recent reports have not really settled the point as to whether Humanin is produced by the *Humanin* gene, the nuclear mitochondrial DNA segment (NUMT) copies, or both, but have concentrated more on the actions of Humanin as a protective agent, especially in AD (Cohen et al., 2015; Hashimoto et al., 2013; Matsuoka, 2015; Tajima et al., 2002), as a retrograde signal peptide passing information about the mitochondrion to the rest of the cell (Lee et al., 2013) and as an agent improving cognition (Murakami et al., 2017; Wu et al., 2017). Synthetic Humanin can now be purchased for research purposes from several companies, and is available with the standard amino acid sequence, or with the 14^th^ amino acid altered from Serine to Glycine, the S14G form, a change which appears to increase the potency of the peptide (Li et al., 2013).

Mitochondria are small organelles found in all developing eukaryotic cells. Each mitochondrion contains a few small rings of double-stranded DNA. Human mtDNA is described as containing 16 569 numbered nucleotide bases, a fairly typical number for a vertebrate, and was the first mitochondrial DNA molecule to be fully sequenced (Anderson et al., 1981). This complete mtDNA sequence was later updated to eliminate the errors (revised Cambridge Reference Sequence [rCRS]; Andrews et al., 1999; Bandelt et al., 2014). The GenBank database (Benson et al., 2005) now holds over 36 000 human mtDNA sequences submitted by research institutes and some private individuals, as well as about 11 000 mtDNA sequences from other vertebrates. The rCRS (GenBank Accession No. NC_012920) describes the mtDNA as having 13 genes for peptides that form part of the OXPHOS system, 22 genes for the production of transfer RNA sequences, a hypervariable control region, several small non-coding sequences situated between the other parts, and most importantly for the present discussion, two genes for the production of RNA sequences (*MT*-*RNR1*, for the RNA 12S molecule & *MT*-*RNR2*, for the RNA 16S molecule) which are structural components used in the building of ribosomes. The *Humanin* gene is located at region 2 633-2 705 in the rCRS and encodes a 24 amino acid peptide. But as this is in the centre of the *MT*-*RNR2* gene, the nucleotide bases of the *Humanin* gene also have the separate function of being a part of the ribosomal RNA 16S molecule.

In this paper simple mining techniques (Yao et al., 2009; Zaki et al., 2007) have been used to look at the *Humanin* gene in the mitochondrial sequences of the human and other vertebrates available in the GenBank database. The study showed that pseudogenization of the *Humanin* gene does not occur in the human, but is a common feature in other vertebrates. Moreover, the study shows the surprise finding that pseudogenization has occurred in a gene in the mtDNA and is not restricted solely to chromosomal genes.

## DATASET AND METHODS

The mitochondrial sequences held in the GenBank database formed the dataset for this study. This database holds about 36 000 different human mtDNA sequences. The individual page on the database for each sequence can be found using a direct link of the form: <uritalic>https://www.ncbi.nlm.nih.gov/nuccore/NC_012920.</uritalic> This particular link connects to the page for the rCRS; and the page for any other sequence can be found by replacing NC_012920 with another Accession No..

A list of the human mtDNA sequences can be found by searching the GenBank database with a query string such as: "homo sapiens" [organism] "complete genome" mitochondrion. Although this list should contain the details of different mtDNA sequences, note in particular that many sequences from the Human Diversity Genome Project are duplicated and in some instances triplicated.

The GenBank database also contains the mitochondrial sequences for approximately 11 000 other vertebrate samples. About 4 000 of these sequences are described as Reference Sequences and have Accession No. in the range NC_000000-NC_999999 (Pruitt et al., 2007). Each Reference Sequence comes from a different species, so the mtDNA sequences available on the GenBank database can be considered as coming from about 4 000 different species of vertebrate.

Unfortunately, GenBank does not give details of the parts of the *MT*-*RNR2* gene in the description accompanying a mtDNA sequence, so it is necessary to identify the *Humanin* sequence by searching for it. However, as the *Humanin* gene is well conserved throughout vertebrates the sequences are able to be identified fairly easily.

Initially, the sequences described in this study were found by visual examination of the FASTA file for each sequence, but subsequently a pattern matching computer program (in Javascript) was developed (Supplementary Program 1, available online). This program requires as its input the *MT*-*RNR2* gene in FASTA format. The 73 bases of the *Humanin* gene as given in the rCRS are then compared by stepping-along the *MT*-*RNR2* gene; and a best-fit is found. A non-matching comparison typically finds about 30 bases in common (i.e., about 40%), but a *Humanin* sequence will match about 50 bases (i.e., about 70%), even for a distant species, and much better for other mammalian species.

The results of this study are presented in three parts: firstly, the variants found in the 36 000+ human mtDNA sequences available for study in the GenBank database; secondly, the *Humanin* sequences found in other vertebrates; and thirdly, the NUMT sequences found in the human and some non-human species.

For clarity, the mtDNA variants are listed in a format of “rCRS allele position derived allele”, instead of the proposed nomenclature of the HVGS (<uritalic>http://varnomen.hgvs.org/</uritalic>). For instance, mtDNA variant T2638C may also be written as m.2638T&gt;C according to HVGS nomenclature, and protein variant P3S written as p.P3S.

## RESULTS

### Variants identified in the human *Humanin* mtDNA sequences

The nucleotide sequence for the *Humanin* gene in the rCRS (GenBank Accession No. NC_012920) was predicted to encode a peptide of 24 amino acid residues, together with a stop codon (MAPRGFSCLLLLTSEIDLPVKRRAX), and this sequence was found in 99.4 % of all the human mtDNA sequences. Data mining of all the human mtDNA genomes in GenBank showed 17 variants are known so far, of which five variants are associated with different mitochondrial haplogroups A2f1 (T2638C, *n*=17, no change in the amino acid sequence), N1b (C2639T, *n*=96, this variant leads to an amino acid change P3S), U6a7a1a (A2672G, *n*=16, this variant changes the amino acid sequence at S14G and produces the well-known potent form of Humanin), G2701A (H13a1a2b, *n*=8, no change in the amino acid sequence), N1a1a (G2702A, *n*=71, this variant changes the amino acid sequence at A24T); and are common enough for them to be used as defining variants in the phylogenetic tree (van Oven & Kayser, 2009) (Supplementary Table 1, available online).

The other 12 variants all occur at low frequencies (1-6 sequences each); and in many of the sequences the presence of a mutation should be considered as 'unverified'. An expanded table of these results is given in the Supplementary Table 1 (available online).

### The *Humanin* sequences found in non-human vertebrates 

Data mining of the non-human vertebrate mtDNA sequences showed *Humanin* sequences are present in all vertebrates. However, many of the sequences show pseudogenization and these sequences are not able to produce functional peptides. A pseudogene is recognizable because of the absence of a start codon, the presence of a premature stop codon, or a deletion/insertion causing a frameshift in the sequence.

**Figure 1:**
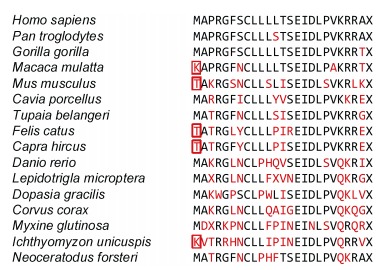
Alignment of Humanin peptide sequences

Amongst our closest relatives are chimpanzees (*Pan troglodytes*) and gorillas (*Gorilla gorilla*), and their *Humanin* sequences are able to produce functional peptide, albeit the sequences differ from that of human by one residue (Figure 1). Some other small mammals showed they should be able to produce functional peptide, e.g., the Guinea pig (*Cavia porcellus*) and Northern tree shrew (*Tupaia belangeri*) (Yao, 2017). However, it was unexpected to find that the Macaques (*Macaca mulatta*) show pseudogenization as there is loss of the start codon.

Most rat and mouse species were shown to have functional genes, but importantly some common mice species (e.g., House mouse (*Mus musculus*)) were found to have undergone pseudogenization. Other common mammals, such as the cat (*Felis catus*) and the goat (*Capra hircus*), were also shown to have undergone pseudogenization. Amongst the bony fishes, the zebrafish (*Danio rerio*) appeared able to produce Humanin, but many other fishes, such as *Lepidotrigla microptera* 'Triglidae' cannot. Some other vertebrate classes may also be able to produce Humanin, such as the lizards (*Dopasia gracilis*) and birds (*Corvus corax*), but whether the peptides are fully functional is uncertain. The most distant vertebrates from ourselves are the lungfish (*Neoceratodus forsteri*), hagfish (*Myxine glutinosa*) and lampreys (*Ichthyomyzon unicuspis*); and whereas hagfish and lampreys show pseudogenization of their *Humanin* sequences, the lungfishes may have retained the ability to make a functional peptide.

The Tuatara (*Sphenodon puctatus*), an ancestor of the snake and found only in New Zealand (Subramanian et al., 2015) is also shown to have a functional gene. But in this species the peptide is one amino acid longer; and it is possible that the gene underwent pseudogenization, only for a later deletion to make the gene functional once again.

Supplementary Table 2 (available online) describes the *humanin* gene in 80 non-human vertebrate species. It is noteworthy that the ability to make the enhanced S14G form of Humanin was identified only in human sequences.

### NUMT sequences in the human, Rhesus macaque, mouse and Golden hamster

As mentioned earlier, the question as to whether NUMT sequences are used to make Humanin is not resolved. But this data mining study showed that in the human there are two NUMT sequences that appear identical to the *Humanin* gene as found in the mtDNA, and a third sequence which is only altered at one amino acid. Also, in the Rhesus macaque (*Macaca mulatta*), the mouse and the Golden hampster (*Mesocricetus auratus*), the *Humanin* gene was shown to have undergone pseudogenization. However, it was also found that a possibly functional NUMT can be found in the chromosomes of each species, suggesting that the pseudogenization may have been a reasonably recent event in the evolutionary past of these species. The details of these sequences are given in the Supplementary Table 3 (available online).

## DISCUSSION

Most of the recent papers dealing with the Humanin peptide have centred on its ability to act as a protective agent, especially in AD (Hashimoto et al., 2001), a retrograde signal peptide (Lee et al., 2013), or as an agent improving cognition (Murakami et al., 2017; Wu et al., 2017), but little has been said about the underlying biology of the *Humanin* gene. In this paper some of the basic points about *Humanin* have been examined by looking at the available sequences of human mtDNA and that of many other vertebrates found in the GenBank database.

In the mtDNA of vertebrates the *Humanin* gene is a gene-within-a-gene as it can be considered as a normal DNA gene and as such has a start-codon, a number of codons which code for amino acids, and finally a stop-codon, which is often represented by a single Thymine nucleotide. However, the bases of the *Humanin* gene are also part of a large RNA structure that is used in the building of ribosomes. Therefore, the *Humanin* gene is both a DNA gene and a RNA gene and as such it can be expected there will be evolutionary pressure to maintain the gene so that it continues to function successfully in both forms. The presumption therefore is that the nucleotide bases of the *Humanin* gene will respond to this evolutionary pressure by showing a low mutation rate. Indeed, the results presented here do suggest that the *Humanin* gene has been strongly conserved throughout vertebrate evolution, and has continued to be functional, for example, in both the human and the lamprey, which have an evolutionary period of separation of over 360 million years (Xu et al., 2016).

In the human, for which there were over 36 000 mtDNA sequences available for study, it appeared that 99.4% of the sequences did not show any mutational differences from that of the rCRS, but in 230 sequences mutations were observed. However, it is likely that the peptide functions normally in most people with mutations, e.g., the mutation C2639T, which is found in people of Haplogroup N1b, changes the 3^rd^ amino acid from a proline to a serine, and a change this close to the end of a peptide usually has little effect. At the other end of the peptide the mutation G2702A changes the 24^th^ amino acid from a glycine to an adenine and similarly can be expected again to have little effect. Indeed, changes to the 3^rd^ and 24^th^amino acids feature prominently in the sequences from other vertebrates.

However of much greater significance is the mutation A2672G, which causes the change of the 14^th^ amino acid from a serine to a glycine. This change S14G is considered to increase the potency of the peptide (Li et al., 2013), and it appears that a few people in the world are able to produce this special form of Humanin naturally. Interestingly, this mutation for the most part is associated with the Haplogroup U6a7a1a, which contains members of an extended Acadian family found in Canada (Secher et al., 2014).

As for other vertebrates, there are mtDNA sequences from over 4 000 species in the GenBank database, and it has been possible to identify the *Humanin* gene unambiguously in all the sequences examined. However, and unexpectedly, many species show pseudogenization of the *Humanin* gene. For example, the human, chimpanzees, gorillas and many other monkeys do have functional genes, but the macaque monkeys, which are widely used in research, do not. Rats, guinea pigs and the northern tree shrew appear able to produce humanin, but some mice, cats and goats do not. So overall, it appears that there is a somewhat random pattern to the evolutionary success of the *Humanin* gene, with it surviving in some species, while undergoing pseudogenization in other apparently closely related species.

However, as with any apparently random pattern, there are features that may well be worthwhile considering further. In the case of the *Humanin* gene there does not appear to be any overt link to the size of an animal, it longevity or its innate intelligence. But it would seem that the presence of a potentially functional *Humanin* gene is commonly found in primates and birds. However, whether this indicates an evolutionary conserved advantage, or is just a feature of the short evolutionary history of these two groups remains to be determined.

The term pseudogene was used for the first time 40 years ago (Jacq et al., 1977) in a study looking at the genome of the African clawed toad, when it was found the genome had multiple copies of a gene. These copies were considered to have no function and were termed pseudogenes. It has since been shown that the genomes of vertebrates contain many different types of pseudogene (Mighell et al., 2000), of which duplication of genes and NUMTs are two of the types. Many pseudogenes are so fragmentary or degraded by subsequent mutations that they are clearly non-functional, but as mentioned earlier, it is possible that some chromosomal copies of the *Humanin* gene are the exception and might still be expressed. Another type of pseudogene formation occurs when a gene is affected by a mutation, or some other process, so that it stops functioning and this process is called pseudogenization. Typically this will occur as the result of loss of the start codon or the introduction of a premature stop codon. Duplication of a gene, and the subsequent loss of functioning of one copy forms another type of pseudogene, but this does not appear to apply to the *Humanin* gene, at least in the human genome (Stark et al., 2017).

The pseudogenization of the *Humanin* gene as detailed here may not solely be an interesting point of evolution, but may also have a significance in studies that use animal models. There have been many studies looking at the effect of Humanin in disease, in particular in AD, which have used animal models, and the results obtained from studies may well have been affected by whether the animals had functional copies of the *Humanin* gene, or pseudogenes. Also, research to find new animal models for many diseases is an important field (Xiao et al., 2017; Yao, 2017), and it would now seem that sequencing of the mtDNA, and in particular the *Humanin* gene, should become routine in any animal under consideration.

Overall, there is still much to be discovered about Humanin. In our own species, and in our close relatives, the peptide appears to be useful; and has been preserved. However, many other species do appear to have kept the ability to produce a Humanin-like peptide, but whether the peptide have the same function as in the human is as yet unknown. This study has also shown there are the many species where pseudogenization has taken place and the *Humanin* pseudogene continues as a sequence of nucleotide bases that form part of the structure of ribosomes.

Why the *humanin* gene has undergone pseudogenization in some species has not been determined. But it would appear that in many species there has been little evolutionary pressure to preserve their ability to make Humanin; and it is only in some species, including ourselves, that it appears to give some evolutionary advantage.

This paper has looked at *Humanin* by reviewing the evidence about the gene available from several sources, and suggests a number of places where experimental work is needed to confirm the findings. It is also possible that future practical work may show that this peptide, in its original or in a synthetic form, may have a therapeutic use in the treatment of conditions such as Alzheimer's disease.

## ACKNOWLEDGEMENTS

The author is very grateful to Yong-Gang Yao (Kunming Institute of Zoology, CAS) for his many helpful comments, especially those concerning the presentation of the material in this paper.
